# Assessment of NLRP3 inflammasome activation in patients with chronic obstructive pulmonary disease before and after lung transplantation

**DOI:** 10.1007/s12026-024-09497-2

**Published:** 2024-05-29

**Authors:** Lada Rumora, Ivona Markelić, Iva Hlapčić, Andrea Hulina Tomašković, Marija Fabijanec, Feđa Džubur, Miroslav Samaržija, Andrea Vukić Dugac

**Affiliations:** 1 Department of Medical Biochemistry and Hematology, Faculty of Pharmacy and Biochemistry, University of Zagreb, Zagreb, Croatia; 2 Clinic for Respiratory Diseases Jordanovac, University Hospital Centre Zagreb, Zagreb, Croatia; 3 Centre for Applied Medical Biochemistry, Faculty of Pharmacy and Biochemistry, University of Zagreb, Zagreb, Croatia; 4 School of Medicine, University of Zagreb, Zagreb, Croatia

**Keywords:** COPD, Lung transplantation, NLRP3, Hsp70, ATP

## Abstract

The interplay between purinergic receptors as well as pattern recognition receptors like Toll-like receptors (TLRs) and NOD-, LRR-, and pyrin domain-containing protein 3 (NLRP3) might have a role in the pathogenesis of chronic obstructive pulmonary disease (COPD). The aim of this study was to determine and compare the concentrations of the damage-associated molecular patterns (DAMPs) heat shock protein 70 (Hsp70) and adenosine triphosphate (ATP), and gene expression of their respective receptors as well as NLRP3 inflammasome-related molecules in the peripheral blood of patients with end-stage COPD before and 1 year after lung transplantation (LT). Lung function was assessed by spirometry and diffusion capacity for carbon monoxide (DLCO). Quantitative polymerase chain reaction (qPCR) was applied for detection of *TLR2*, *TLR4*, *P2X7R*, *P2Y2R*, *IL1B*, *CASP1*, and *NLRP3* expression. High-sensitivity ELISA kits were used for extracellular (e) Hsp70 and IL-1β, and luminescence assay for eATP measurements. Concentrations of eHsp70 and eATP as well as IL-1β were significantly increased in the plasma of end-stage COPD patients and significantly decreased after LT. In addition, *TLR4*, *P2Y2R*, *IL1B*, *CASP1*, and *NLRP3* expression was up-regulated in COPD patients before LT, while it was significantly suppressed after LT. In conclusion, it could be assumed that NLRP3 inflammasome is activated in the peripheral blood of end-stage COPD patients and that eHsp70 and eATP could be responsible for its activation through triggering their receptors. On the other hand, previously enhanced pro-inflammatory reactions seem to be suppressed to the healthy population levels in lung recipients without allograft rejection.

**Supplementary Information:**

The online version contains supplementary material available at 10.1007/s12026-024-09497-2.

## Introduction

Chronic obstructive pulmonary disease (COPD) is historically the most common indication for lung transplantation in adults, and according to the latest International Society for Heart and Lung Transplantation (ISHLT) registry, it represents 26.5% of all lung transplantations [1].

COPD is the third leading cause of death worldwide, and its burden is ever-increasing [2]. Global Initiative for Chronic Obstructive Lung Disease (GOLD) recognizes and defines COPD as a heterogeneous lung condition characterized by chronic respiratory symptoms due to airways and/or alveolar abnormalities that cause persistent, often progressive, airflow obstruction [3]. Chronic inflammation is associated with COPD development with possible altered inflammatory responses in both local airways and systemic compartments. It was suggested that multiprotein complexes inflammasomes might be involved in the pathogenesis of COPD, especially the NOD-, LRR-, and pyrin domain-containing protein 3 (NLRP3) inflammasome [4]. NLRP3 inflammasome consists of a sensor NLRP3 which belongs to the intracellular pattern recognition receptors (PRRs), an adaptor (apoptosis-associated speck-like protein containing a caspase activation and recruitment domain (ASC)) and an effector (caspase-1). It is activated by various pathogen-associated molecular patterns (PAMPs) and/or damage-associated molecular patterns (DAMPs). The classical canonical inflammasome pathway requires two signals for its activation. In the priming step PAMPs or DAMPs bind to their PRRs, often Toll-like receptor (TLR) 2 and TLR4, and induce expression of inflammasome components as well as inactive precursors of interleukin (IL)-1β and IL-18. In the activation step various stimuli, including DAMPs, indirectly trigger NLRP3 inflammasome assembly and activation of caspase-1, leading to secretion of mature IL-1β and IL-18 and an inflammatory form of cell death called pyroptosis. In non-canonical pathway, inflammasome activation is triggered by caspase-11 in mice and caspase-4/5 in humans that directly recognize intracellular lipopolysaccharide (LPS), and this inflammasome activation mode also leads to the release of pro-inflammatory cytokines and pyroptosis. Contrary to this, the alternative inflammasome pathway requires only one signal and does not provoke pyroptosis. Apart from NLRP3, several other inflammasomes have been discovered, including NLRP1, NLRC4, and AIM2 [5, 6].

In our previous studies, we used various bronchial epithelial and monocytic cell lines and primary cells that were representative as potential COPD airway and systemic in vitro models, and we suggested the involvement of two DAMP molecules, namely heat shock protein 70 (Hsp70) and adenosine triphosphate (ATP), in NLRP3 inflammasome activation [7, 8].

ATP is a purine nucleotide that acts as the principal immediate donor of free energy in most cellular energy-requiring processes such as motion, active transport, and biosynthesis [9]. ATP is an important signaling molecule when present in the extracellular milieu. There it originates from necrotic cells but is also released from intact cells through exocytosis and several types of membrane transporters or channels [10].

Released ATP subsequently triggers autocrine and/or paracrine activation of purinergic receptors. ATP receptors belong to the P2 nucleotide receptor family, which is further subdivided into P2X (ionotropic or cation-permeable ATP-gated ion channels) and P2Y (metabotropic G protein-coupled receptors). To date, seven P2X receptors have been discovered (P2X_1 – 7_) with ATP being their only known physiological agonist. On the other hand, there are eight P2Y receptor members (P2Y_1/2/4/6/11 – 14_) that might be activated by several nucleotides [11].

Regarding NLRP3 inflammasome activation, P2X7R is recognized as one of its most potent inducers [12], but the role of some other members of the purinergic receptor family in this process has also been implicated, such as for P2Y2R, which has a similar affinity for ATP and uridine triphosphate (UTP) [10, 13–15].

Hsp70 is a member of the heat shock protein family that is mainly found intracellularly where it exerts its function as molecular chaperone. When needed, its expression might be induced by stressful stimuli [16]. In various pathological conditions, Hsp70 might be increasingly released from a cell in a lytic (necrosis) and non-lytic manner, and then it acts like DAMP and alarms the immune system by activation of its specific PRRs, such as TLR2 and TLR4. Excessive TLR activation disrupts the immune homeostasis through the sustained production of pro-inflammatory cytokines and chemokines, which can lead to the development and/or progression of various diseases [17]. Numerous data suggest an important role of TLRs in the immunoregulation of the inflammatory process in COPD, with TLR2 and TLR4 being particularly important [18–21].

Currently, there is still no specific, curative treatment for COPD. All the standard treatments can achieve slowing of lung function decline, but for many patients with end-stage lung disease, lung transplantation plays a vital role as the only viable treatment option when the other therapeutic alternatives have been exhausted.

There are limited and/or conflicting information on inflammatory responses in COPD associated with NLRP3 inflammasome activation, especially in the systemic compartment. In COPD patients, NLRP3 is overexpressed in the lung and its expression correlates with airflow obstruction [22]. Wang et al. evaluated a possible correlation between NLRP3 inflammasome activation and the risk of acute exacerbation of COPD (AECOPD). They found that the expression levels of NLRP3 inflammasome components in peripheral blood mononuclear cells (PBMCs) and bronchial tissues from patients with AECOPD were significantly higher than those in smokers without lung diseases [23]. Similar results have been shown in our previous studies where we found elevated levels of extracellular ATP (eATP) and extracellular Hsp70 (eHsp70), and we also suggested NLRP3 inflammasome activation in COPD patients [4, 24, 25]. We demonstrated increased *IL1B*, *NLRP3*, and *CASP1* gene expression as well as the plasma concentration of IL-1β in the peripheral blood of COPD patients compared to the healthy controls, but our patients were in a stable phase of the disease [4]. Other studies that looked at IL-1β in peripheral blood found that COPD patients have also higher plasma concentrations of IL-1β than healthy controls [26]. On the contrary, in the study by Kleniewska et al., IL-1β was elevated in the induced sputum of COPD patients, although its serum concentration was the same as that of healthy subjects [27]. In the study by Damera et al., concentration of IL-1β was increased in the sputum and serum of COPD patients when compared to healthy controls [28]. Regarding non-canonical inflammasome pathway, De Falco et al. demonstrated that IL-18 and IL-33 release from combustion-generated ultrafine particles (UFPs) treated PBMCs of unstable/exacerbated COPD patients correlated with caspase-4 release, and this effect was not observed in stable COPD-derived PBMCs. They suggested that combustion-generated UFPs induce the release of caspase-4-dependent inflammasome from PBMCs of COPD patients compared with healthy subjects [29]. Colarusso et al. reported up-regulation of the AIM2 inflammasome pathway in PBMCs of COPD patients during exacerbations but not during stable periods [30].

However, so far, there are no available data comparing the activation state of the NLRP3 inflammasome and the levels of its possible agonists in COPD patients before and after lung transplantation. Therefore, this study investigated the plasma concentration of the DAMPs eHsp70 and eATP and the gene expression of their receptors TLR2 and TLR4 as well as P2X7R and P2Y2R, respectively. We also measured the concentration of the cytokine IL-1β and its mRNA levels as well as the mRNA levels of NLRP3 inflammasome components NLRP3 and caspase-1 in patients with end-stage COPD before lung transplantation and 1 year after lung transplantation.

## Materials and methods

### Study population

This study is the final part of a larger research that lasted for 4 years (basic characteristics of research participants are presented in Supplementary Table 1. During that period 109 COPD patients in the stable phase of the disease were voluntarily recruited at the Clinical Department for Lung Diseases Jordanovac, University Hospital Centre Zagreb (Zagreb, Croatia), as we previously described [21, 24, 25]. They signed an informed consent for scientific research and agreed to volunteer. Ethical Committee of University Hospital Centre Zagreb and Ethical Committee for Experimentation of Faculty of Pharmacy and Biochemistry, University of Zagreb (Zagreb, Croatia) approved the study (Approval Protocol Numbers: 02/21/JG and 251-62-03-14-78, respectively). Briefly, COPD diagnosis was confirmed by pulmonology specialists according to the GOLD guidelines. The patients were classified into GOLD grades 2 (*n* = 39), 3 (*n* = 36), and 4 (*n* = 34) reflecting the severity of airflow obstructions. In addition, data on symptoms severity and history of exacerbations were used to classify COPD patients into GOLD groups A (*n* = 14), B (*n* = 63), C (*n* = 0), and D (*n* = 32). According to the latest GOLD report, groups C and D are merged into a single group termed E. However, in our research, none of the recruited patients were classified into group C, and this group of patients is often very rare, as patients who do not have many symptoms usually are not frequent exacerbators.

At the end of the patients’ follow up, five patients with end-stage COPD (all in GOLD 4 stage and GOLD D (now E) group, all ex-smokers) underwent bilateral lung transplantation in the Vienna General Hospital (Allgemeines Krankenhaus der Stadt Wien–AKH). Immunosuppressive therapy consisted of induction with alemtuzumab, followed by maintenance with tacrolimus and corticosteroids (5 mg prednisone) during first year after lung transplantation (LT). It is important to emphasize that during this first post-operative year recipients did not develop any significant complications. Their measured parameters before LT and 1 year after LT were compared as a part of this current study.

### Assessment of lung function

Airflow limitation was diagnosed by spirometry on a MasterScreen Pneumo Spirometer (Jaeger, Wurzburg, Germany), and airflow obstruction was confirmed if forced expiratory volume in the first second (FEV_1_)/ forced vital capacity (FVC) was lower than 0.70 after three acceptable measurements. In addition, diffusion capacity for carbon monoxide (DLCO) was measured on MasterScreen PFT Pro (Jaeger, Wurzburg, Germany), as we previously described [24].

### Measurement of eHsp70

Blood samples were collected by venepuncture of a large antecubital vein after overnight fasting from 7 a.m. to 9 a.m. into tubes with anticoagulant tripotassium ethylenediaminetetraacetic acid (K3 EDTA) (Greiner Bio-One, GmbH, Kremsmünster, Austria). After centrifugation at 1000× *g* for 15 min at 4 °C, plasma was separated and stored at −80 °C until eHsp70 determination. The AMP’D HSP70 high-sensitivity ELISA kit (Enzo Life Science, Farmingdale, NY, USA) was used for the eHsp70 concentration measurement, and the manufacturer’s protocol was followed. As recommended, EDTA plasma samples were minimally diluted in a 1:4 ratio with assay buffer to remove matrix interference. The concentration of eHsp70 was determined at 495 nm using the SpectraMax i3x multi-mode microplate reader (Molecular Devices, San Jose, CA, USA). The eHsp70 concentration in the samples was calculated using a four-parameter logistic curve fit in OriginPro version 9.0 software (OriginLab Corporation, Northampton, MA, USA).

### Measurement of eATP

ATP concentration was determined using the ATPlite assay (Parking Elmer, Waltham, Massachusetts, USA), mostly by following the manufacturer’s instructions, but with some modifications. We added the mammalian cell lysis solution, which is a part of the assay kit, into each plasma sample to inactivate ATPases. In addition, plasma samples were diluted in a 1:10 ratio with water. Luminescence was measured by the SpectraMax i3x multi-mode microplate reader (Molecular Devices, San Jose, CA, USA). Later, a four-parameter logistic curve fit was used for calculating ATP concentrations using OriginPro version 9.0 software program (OriginLab Corporation, Northampton, Massachusetts, USA).

### Measurement of IL-1β

IL-1β concentrations were measured in EDTA plasma using the Quantikine HS ELISA human IL-1β/IL-1F2 immunoassay (R&D Systems, Minneapolis, MN, USA), according to the manufacturer’s protocol. The concentration of IL-1β was measured at 450 nm using the SpectraMax i3x multi-mode microplate reader (Molecular Devices, San Jose, CA, USA). IL-1β concentration was calculated by a four-parameter logistic curve fit in OriginPro version 9.0 software (OriginLab Corporation, Northampton, MA, USA).

### Gene expression analysis

Tubes containing K3 EDTA as an anticoagulant (Greiner Bio-One, Kremsmünster, Austria) were used for the buffy coat extraction. The TRIzol/chloroform method [31], after centrifugation at 3500 rpm for 10 min at + 4 °C, was used for the total RNA isolation from the buffy coat. The quality of RNA was assessed by the ratio of 260/280 nm using the microvolume spectrophotometer Nanodrop 8000 (Thermo Fischer Scientific, Wilmington, USA). When the ratio was suitable (1.9–2.1), we performed cDNA synthesis by reverse transcription based on polymerase chain reaction (PCR) using RevertAid First Strand cDNA Synthesis Kit (Thermo Fischer Scientific, Waltham, MA, USA) by GeneAmp PCR System 9700 (Applied Biosystems, Foster City, USA) with PCR conditions being 5 min at 25 °C, 60 min at 42 °C and 5 min at 70 °C [32]. The assessment of *TLR2*, *TLR4*, *P2X7R*, *P2Y2R*,* IL1B*, *NLRP3*, and *CASP1* gene expressions were performed using TaqMan Gene Expression Assays (Hs02621280_s1 for *TLR2*, Hs00152939_m1 for *TLR4*, Hs00175721_m1 for *P2X7R*; Hs04176264_s1 for *P2Y2R*; Hs01555410_m1 for *IL1B*, Hs00918082_m1 for *NLRP3*, Hs00354836_m1 for *CASP1*, Applied Biosystems, Foster City, USA) and TaqMan Universal Master Mix (Applied Biosystems, Foster City, USA) by following the guidelines of the manufacturer. The cycling parameters for qPCR were 2 min at 50 °C, 10 min at 95 °C, followed by 40 cycles of 15 s at 95 °C and 60 °C for 1 min, using the 7500 Real-Time PCR System (Applied Biosystems, Foster City, USA). Data were normalized using gene expression of beta-2-microglobulin (*B2M*) and peptidylprolyl isomerase (*PPIA*) (Hs99999907_m1 for *B2M*, Hs99999904_m1for *PPIA*; Applied Biosystems, Foster City, USA) as the endogenous controls. Also, a randomly selected sample was included as a calibrator in each plate. The relative expression of target genes was performed using the 2^−∆∆Ct^ comparison method [33].

### Statistics

As all data failed the Kolmogorov–Smirnov normality test and the tested group of interest was small, the results were shown as a median with corresponding interquartile range.

Non-parametric Wilcoxon signed rank test was used to test the differences between patients’ parameters before and after LT. Analysis was performed in MedCalc statistical software version 17.9.2. (MedCalc Software, Ostend, Belgium). Results were statistically significant if *P* < 0.05.

## Results

### Lung function parameters and health impairment questionnaires

Our longitudinal research included 109 COPD patients in the stable phase of the disease, with 34 patients in GOLD 4 stage and 32 patients in GOLD D group. At the end of their follow up, five patients with end-stage COPD underwent bilateral lung transplantation. Their median age was 53 (51–62) years. There were two men and three women. All lung recipients were ex-smokers for several years before transplantation. Importantly, none of them developed acute or chronic rejection during 1-year monitored post-transplantation period.

As expected, all relevant spirometry parameters for assessing the severity of airflow obstructions were significantly increased 1 year after transplantation (Table 1).

**Table 1:** Lung function parameters and health impairment questionnaires’ scores obtained from COPD patients before and after lung transplantation

Parameter	Before LT	After LT	*P*-value
FEV_1_ (L)	0.59 (0.47–0.75)	3.22 (2.02–3.59)	*P* = 0.002
FVC (L)	1.81 (1.28–2.03)	3.57 (2.36–5.09)	*P* = 0.014
FEV_1_ (% pred.)	21 (18–24)	106 (81–114)	*P* < 0.001
FEV_1_/FVC (%)	35 (28–43)	74 (69–91)	*P* = 0.001
DLCO	25 (19–27)	58 (47–74)	*P* = 0.024
mMRC	3.00 (3.00–3.00)	0.00 (0.00–1.25)	*P* = 0.041
CAT	23.00 (22.00–26.25)	0.00 (0.00–2.50)	*P* = 0.035
SGRQ-C	70.90 (60.08–74.90)	4.77 (1.59–12.93)	*P* = 0.015

Data were analyzed by Wilcoxon signed rank test and shown as median with interquartile range. Results were statistically significant when *P* < 0.05

Abbreviations: *FEV*_1_ forced expiratory volume in the first second, *FVC* forced vital capacity, *DLCO* diffusion capacity for carbon monoxide, *mMRC* modified Medical Research Council, *CAT* COPD Assessment Test, *SGRQ-C* St. George’s Respiratory Questionnaire for COPD patients

In addition, DLCO, which was used for the assessment of the diffusion properties of the alveolar−capillary membrane, was also significantly elevated after LT.

COPD patients completed three different questionnaires that were used for the assessment of patients’ symptoms and quality of life: modified Medical Research Council (mMRC) dyspnea scale, COPD Assessment Test (CAT), and St. George’s Respiratory.

Questionnaire for COPD patients (SGRQ-C). According to their self-reported answers, their health status significantly improved after transplantation.

### Decrease in concentrations of eHsp70, eATP, and IL-1β after LT

Firstly, we measured the concentrations of eHsp70, eATP, and IL-1β when the patients were in the stable phase of end-stage of COPD. Secondly, we measured the same parameters in EDTA plasma 1 year after LT.

As shown in Table 2, the concentrations of DAMP molecules eHsp70 and eATP significantly decreased after transplantation (*P* = 0.010 and *P* < 0.001, respectively). Similarly, levels of cytokine IL-1β were lower in the post-transplantation period (*P* < 0.001).

**Table 2:** Concentrations of eHsp70, eATP, and IL-1β in plasma of COPD patients before and after lung transplantation

Parameter	Before LT	After LT	*P*-value
eHsp70 (ng/mL)	3.10 (1.78–3.56)	0.39 (0.25–0.69)	*P* = 0.010
eATP (µM)	3.72 (3.34–4.02)	0.85 (0.56–1.21)	*P* < 0.001
IL-1β (pg/mL)	15.68 (8.12–26.34)	0.42 (0.19–1.02)	*P* < 0.001

Data were analyzed by Wilcoxon signed rank test and shown as median with interquartile range. Results were statistically significant when *P* < 0.05

Abbreviations: *eHsp70* extracellular heat shock protein 70, *eATP* extracellular adenosine triphosphate, *IL-1β* interleukin 1β

### Gene expression encoding eHsp70 and eATP receptors as well as IL-1β and NLRP3 inflammasome components

Finally, we explored the relative gene expression of two eHsp70 receptors (*TLR2* and *TLR4*), two eATP receptors (*P2X7R* and *P2Y2R*), *IL-1B*, and NLRP3 inflammasome components (*NLRP3* and *CASP1*) in the systemic compartment of COPD patients before and after lung transplantation.

**Fig. 1:**
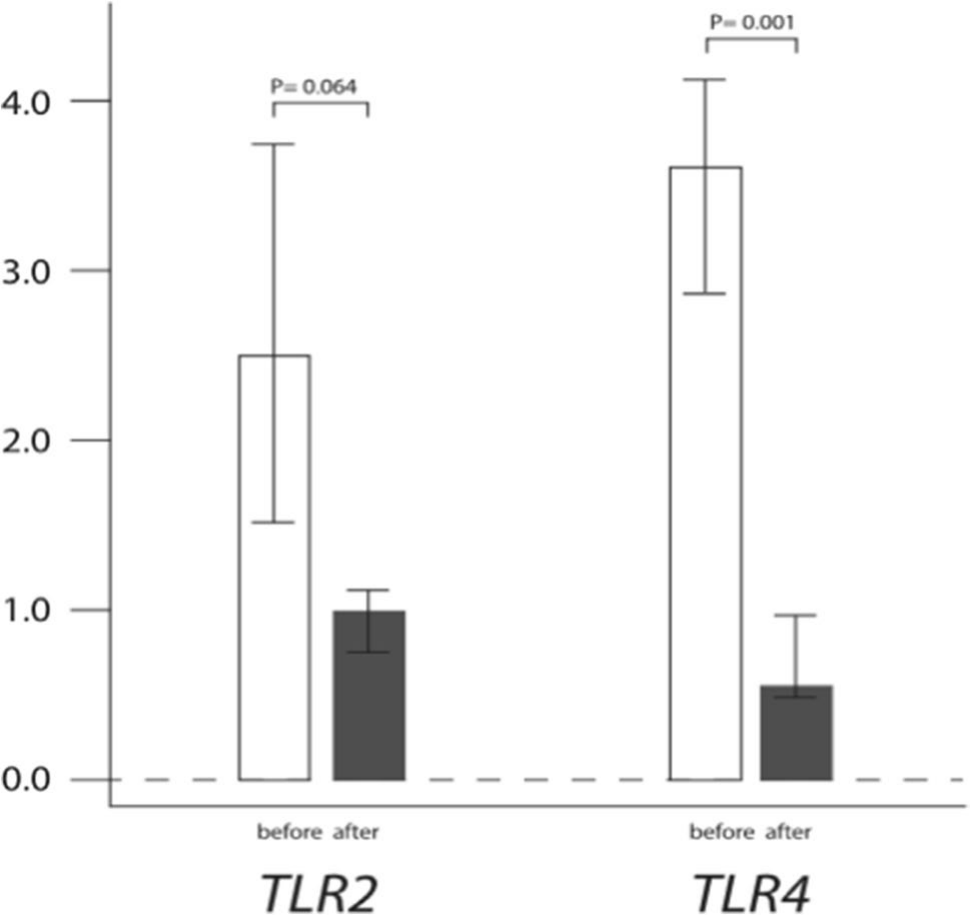
Relative gene expression of *TLR2* and *TLR4* in end-stage COPD patients before and after lung transplantation. Results were presented as a median and interquartile range

As for eHsp70 receptors, *TLR4* gene expression was significantly down-regulated after LT (*P* = 0.001). However, although the mRNA levels of *TLR2* tended to decrease in post-transplantation period, the results were not statistically significant (*P* = 0.064) (Figure 1).

**Fig. 2:**
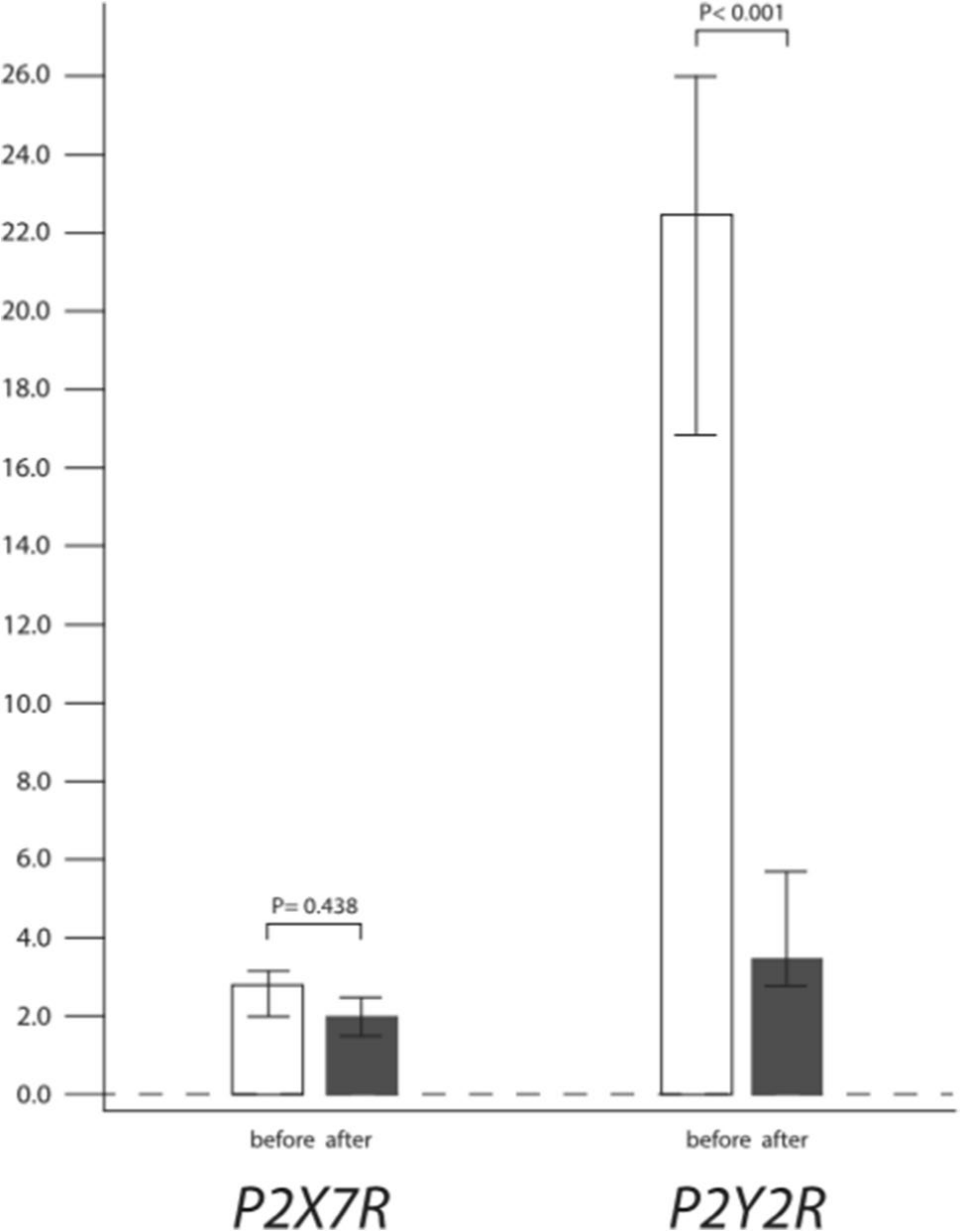
Relative gene expression of *P2X7R* and *P2Y2R* in end-stage COPD patients before and after lung transplantation. Results were presented as a median and interquartile range

As for eATP receptors, similar mRNA levels of *P2X7R* were detected before and after LT (*P* = 0.438), while *P2Y2R* gene expression was significantly lower after LT (*P* < 0.001) (Figure 2).

Lastly, gene expressions of *IL-1B* as well as *NLRP3* and *CASP1* were significantly down-regulated in patients 1 year after transplantation (*P* = 0.005, *P* = 0.009, and *P* = 0.014, respectively) (Table 3). Abbreviations:* IL1B* interleukin-1𝛽, *NLRP3* NOD-, LRR- and pyrin domain-containing protein 3, *CASP1* caspase-1

**Table 3:** Relative expression of genes encoding IL-1β and NLRP3 inflammasome components

Parameter	Before LT	After LT	*P*-value
*IL1B*	2.64 (2.19–3.18)	0.81 (0.48–0.94)	*P* = 0.005
*NLRP3*	4.74 (3.62–7.24)	1.54 (1.44–2.19)	*P* = 0.009
*CASP1*	2.27 (2.00–2.71)	1.16 (0.52–1.26)	*P* = 0.014

Data were analyzed by Wilcoxon signed rank test and shown as median with interquartile range. Results were statistically significant when *P* < 0.05

## Discussion

In this study, we assessed some specific innate immunity-related molecules in patients with end-stage COPD and in the same individuals 1 year after bilateral lung transplantation. We found significantly decreased concentrations of DAMP molecules eHsp70 and eATP as well as cytokine IL-1β in plasma at the post-transplantation time point compared to the pre-transplantation time point. We also detected suppression of TLR4, P2Y2R, IL1B, CASP1, and NLRP3 gene expression after LT.

Previous research with lung transplant recipients were focused on detection of pro-inflammatory parameters in conditions of acute allograft rejection (mostly as a result of ischemia-reperfusion injury (IRI)) and chronic lung allograft dysfunction (CLAD). In those conditions, damaged and/or necrotic cells could release increased amounts of various DAMPs which can further enhance immune responses by triggering the activation of various PRRs.

Ibrahim et al. demonstrated that dogs treated with apyrase, used to hydrolyze ATP, had better protection from IRI, with better lung function and less pulmonary edema [34]. The same authors also showed that human lung recipients with moderate to severe CLAD had increased eATP concentration in bronchoalveolar lavage (BAL) fluid when compared to those without this injury [34]. In addition, eATP levels were increased in mouse lung allografts and become reduced when a nonselective inhibitor of purinergic receptors or a specific P2X7R inhibitor was applied and that was accompanied with diminished acute rejection and prolonged survival of mouse lung allografts [35].

As for Hsp70, only its intracellular form was detected in lung allotransplantation. Its synthesis was stimulated by IRI, which is suggested to have a protective role on transplanted organs [36, 37].

The role of TLRs, including TLR2 and TLR4, in lung transplantation has been explored in both human and animal models. Garantziotis et al. suggested that local activation of pulmonary innate immunity by lipopolysaccharide (LPS) and its receptor TLR4 is sufficient for the development of obliterative bronchiolitis (OB) after lung transplantation in recipient mice [38]. In addition, Kastelijn et al. found that specific TLR2, TLR4, and TLR9 polymorphisms are associated with a higher incidence of CLAD [39]. Therefore, TLRs might modulate CLAD development.

The role of the NLRP3 inflammasome in lung allograft rejection was also investigated. Cantu et al. intended to find important pathways involved in transplant lung injury by studying changes in gene expression in BAL fluid of patients with grade 3 primary graft dysfunction (PGD) compared to controls without PGD. They found significant up-regulation in eight gene sets, and most of them were directly related to the NLRP3 inflammasome. Among individual transcripts, IL-1β and NLRP3 were the most up-regulated in grade 3 PGD compared to controls, while TLR4 was also among the highest ones [40]. In a mouse model of CLAD, NLRP3 inflammasome activation was shown to be significantly increased after tracheal transplantation. Still, OB lesions ameliorated after the application of a specific inhibitor of the NLRP3 inflammasome, suggesting that NLRP3 activation is implicated in the progression of CLAD in allograft tissue [41].

However, it must be emphasized that our study subjects, who were in the end-stage of COPD before lung transplantation, did not develop any signs of CLAD through the 1-year monitored post-transplantation period. To the best of our knowledge, there are no studies that compared the aforementioned parameters in the peripheral blood of COPD patients before and after LT, including the patients who did not experience allograft rejection.

The only similar study design that we could find was the one of McElvaney et al., who demonstrated significantly decreased concentrations of IL-1β, that might reflect the NLRP3 inflammasome activation state, in BAL fluid after double lung transplantation, but this was assessed in patients with cystic fibrosis, and not COPD [42].

In this study, concentrations of eHsp70 were high in the plasma of end-stage COPD patients before transplantation of the lungs. Only a few studies measured blood levels of eHsp70 in patients with COPD [25, 43–45]. In our previous research, which included greater number of COPD patients in the stable phase of the disease and their age- and sex-matched controls, we found that eHsp70 was increased in patients’ peripheral blood compared to healthy subjects, and this increase was associated with severity of airflow limitation as well as symptoms burden and exacerbation history [25]. Here, patients with end-stage COPD before LT had eHsp70 level even significantly higher than patients from our former COPD cohort that were in the GOLD 4 stage or GOLD D group (3.10 (1.78–3.56) ng/mL vs. 1.26 (1.07–1.65) or 1.14 (0.98–1.47) ng/mL, respectively). On the other hand, post-transplantation concentrations of eHsp70 were similar to those in the healthy control group from the previous study (Supplementary Table 2) [25].

Some studies related to COPD reported alterations in expression of eHsp70 receptors TLR2 and TLR4. Haw et al. showed that cigarette smoke (CS)-induced airway fibrosis was not modulated in TLR2^−^^/−^ mice but was suppressed in TLR4^−/−^ mice compared to CS-exposed wild-type controls. In addition, CS-induced emphysema-like alveolar enlargement, apoptosis, and impaired lung function were enhanced in TLR2^−/−^ mice and were decreased in TLR4^−/−^ mice [19]. Also, differential expression of TLR2 and TLR4 was observed in the airways of COPD patients [19, 46].

However, there are scarce data on TLR2/4 expression in circulation although the inflammatory process in COPD is both local and systemic. Previously, we found increased TLR2 expression in the peripheral blood of COPD patients [21], while in this study, end-stage COPD patients had increased mRNA level of TLR4 before LT, but TLR4 was down-regulated in lung recipients and its relative expression level was similar to that one in healthy control group (Supplementary Table 3) [21].

It was suggested that both the excessive activation and the suppression of TLR2/4 might be involved in COPD pathogenesis, and that modulation of TLR2 and/or TLR4 expression might become relevant in the future COPD therapeutic regime [18, 19].

Similar to eHsp70, concentrations of eATP were increased in end-stage COPD patients of this study. Elevated eATP concentrations were found in BAL fluid of COPD patients [23], and in supernatants of various bronchial epithelial and monocytic cell lines and primary cells that were representative as potential COPD airway and systemic in vitro models [7, 8]. Only our previous study measured eATP levels in the peripheral blood of COPD patients and found that they were elevated compared to healthy controls. Those concentrations of eATP were associated with airflow obstruction grades, and with symptoms and exacerbation history in previous year [24]. In this study, concentrations of eATP in end-stage COPD patients were slightly higher compared to those in patients from our former COPD cohort that were in GOLD 4 stage or GOLD D group (3.72 (3.34–4.02) µM vs. 3.19 (2.86–3.67) or 3.08 (2.07–3.57) µM, respectively) [25]. On the other hand, eATP levels decreased after LT to the levels similar to those in healthy subjects (Supplementary Table 2) [25].

Physiologically, ATP’s extracellular concentration is maintained very low by ectonucleotidases, mainly by the cluster of differentiation (CD) 39 and CD73. However, under certain pathological conditions, such as infection or inflammation, certain mediators induce the release of ATP and/or down-regulate ectonucleotidases’ CD39 and/or CD73 levels [9]. It remains unclear whether the findings from this study are due to a stimulation of ectonucleotidases or a suppression of ATP release or both in lung transplant recipients, and this would be interesting to explore in our future research.

When we measured gene expression of eATP receptors, we found that *P2X7R* mRNA levels were similar in individuals before and after LT, but *P2Y2R* expression was significantly increased in end-stage COPD patients. Also, in our previous study, *P2X7R* expression did not differ between patients with COPD and healthy subjects, while P2Y2R expression was significantly up-regulated in COPD patients’ peripheral blood [24].

Regarding P2Y2R, it was shown that wood smoke particulate matter treatment of human bronchial epithelial cell line 16HBE increased ATP secretion, activated caspase-1/IL-1β/IL-18 signaling pathway, and induced pyroptosis through P2Y2R/P2Y7R-dependent mechanisms [14]. In addition, de la Rosa et al. found an association between P2Y2R and IL-1β concentration and between P2Y2R and *IL1B* gene expression in murine resident peritoneal macrophages when they applied the specific P2Y2R inhibitor. They also detected dose-dependent release of IL-1β, with ATP peaking effect at 20 µM [15].

On the other hand, it was shown that among homotrimeric P2X receptors, P2X7R is the least sensitive member that requires higher ATP concentrations for its activation (in hundred micromolar range) [11].

Therefore, eATP is able to activate various purinergic receptors on target cells, and this might be dependent on its concentration. It seems that higher ATP concentrations are necessary to activate P2X7R (in this scenario ATP could originate from necrotic cells and it accumulates locally), while lower concentrations (like those present in peripheral blood) might activate P2Y2R.

Activation of P2X7R or P2Y2R as well as TLR2 or TLR4 could be associated with NLRP3 inflammasome activation [5, 6]. In this study, we demonstrated an increased plasma concentration of the cytokine IL-1β, which might represent the end-point parameter for activation of NLRP3 inflammasome, and up-regulated *IL1B* as well as *NLRP3* and *CASP1* gene expression in patients with end-stage COPD in comparison with the levels of the same parameters determined 1 year after lung transplantation. There are only a few studies regarding NLRP3 inflammasome in the systemic compartment of COPD patients. Increased *IL1B*, *NLRP3*, and *CASP1* mRNA levels as well as concentration of IL-1β in peripheral blood were shown in acute exacerbation of COPD [47]. Similar results were obtained in our previous study with COPD patients in the stable phase of the disease [4]. In this study, the concentrations of IL-1β were higher in end-stage COPD patients than in patients from our previous COPD cohort, and were only slightly higher after lung transplantation than in healthy individuals (Supplementary Table 2) [4].

Along the lines of those results, *IL1B*, *NLRP3*, and *CASP1* relative gene expression was higher in COPD patients from this study before they underwent LT in comparison to the levels obtained in our former study with COPD patients (Supplementary Table 4). On the other hand, these expressions were quite similar in lung recipients and healthy subjects [4]. Therefore, it could be speculated that NLRP3 inflammasome is activated in COPD patients, and after successful lung transplantation without significant acute and chronic rejection, its activation is suppressed by finely tuned immune responses that are involved in the regulation of tissue homeostasis and host protection.

The results of this study should be interpreted considering several limitations. The most important one is the small number of participants that were included in the study. In addition, there are no previous studies designed as our present study, so the discussion is based on the hypothesis that study subjects are similar to healthy controls after lung transplantation, which was confirmed by the values of spirometry parameters and DLCO as well as by the scores of different questionnaires used to assess the patients’ quality of life. To further confirm this assumption, we also compared lung recipients with healthy subjects from our former study. Finally, it should be highlighted that all transplant recipients are taking immunosuppressive therapy which might affect the immune system and its responses.

Glucocorticoids, as a part of an immunosuppressive regimen, regulate the immune response at both the cellular and transcriptional levels. At the transcriptional level, glucocorticoids can decrease the inflammatory response through reduced production of cytokines, IL-1, IL-2, IL-6, interferon (IFN)-γ, and tumor necrosis factor (TNF)-α [48]. At the cellular level, glucocorticoids can induce apoptosis of T lymphocytes, neutrophils, basophils, and eosinophils in order to reduce inflammation [49]. By inhibiting the nuclear factor κB (NF-κB) pathway, by direct or indirect interaction with this transcription factor, glucocorticoids also efficiently suppress inflammation by regulating the expression of IL-1β [48]. Moreover, corticosteroids are well-known to induce IL-1 receptor antagonist (IL-1ra) transcription and therefore it is likely that the administration of corticosteroids was able to reduce IL-1β release although there are some contradicted data about corticosteroids affecting IL-1ra [50, 51]. On the other hand, tacrolimus exerts its immunosuppressive effect by reducing interleukin-2 production and IL-2 receptor expression, leading to a reduction in T-cell activation [51]. Hence, the use of corticosteroids and tacrolimus may be the cause of the lowered concentrations of DAMP molecules and the cytokine IL-1β, as well as the reduced TLR4, P2Y2R, IL1B, CASP1, and NLRP3 gene expression following LT.

The Authors should discuss the results and how they can be interpreted from the perspective of previous studies and of the working hypotheses. The findings and their implications should be discussed in the broadest context possible. Future research directions may also be highlighted.

## Conclusions

In conclusion, it could be suggested that the NLRP3 inflammasome is activated in the peripheral blood of end-stage COPD patients and that eHsp70 and eATP could be responsible for its activation through triggering their respective receptors. On the other hand, in lung recipients without allograft rejection previously amplified pro-inflammatory responses seem to be reduced to the levels present in healthy organisms.

Activation of PRRs (TLR2/4 and NLRP3) and purinergic receptors (P2X7R or P2Y2R) by DAMPs can provoke and enhance lung inflammation and remodeling as well as systemic inflammation, which might contribute to the development and progression of COPD. Therefore, both various DAMPs and innate immune receptors could become an attractive diagnostic and/or therapeutic target for end-stage COPD in the future. However, for now, lung transplantation is a limited but available option for carefully selected patients with severe COPD.

## Electronic supplementary material

Below is the link to the electronic supplementary material.

Supplementary Material 1

## Data Availability

All data will be made available upon request to the authors.

## Abbreviations

ATP: Adenosine triphosphate
ASC: Apoptosis-associated speck-like protein containing a caspase activation and recruitment domain
AECOPD: Acute exacerbation of COPD
BAL: Bronchoalveolar lavage
CASP1: Caspase-1
CAT: COPD Assessment Test
CD: Cluster of differentiation
CLAD: Chronic lung allograft dysfunction
COPD: Chronic obstructive pulmonary disease
DAMPs: Damage-associated molecular patterns
DLCO: Diffusion capacity for carbon monoxide
eATP: Extracellular adenosine triphosphate
eHsp70: Extracellular heat shock protein 70
FEV_1_: Forced expiratory volume in the first second
FVC: Forced vital capacity
GOLD: Global initiative for chronic obstructive lung disease
Hsp70: Heat shock protein 70
IL-1β: Interleukin 1β
IL1Rs: Interleukin-1 receptors
IRI: Ischemia-reperfusion injury
LPS: Lipopolysaccharide
LT: Lung transplantation
mMRC: Modified Medical Research Council dyspnea scale
NF-kB: Nuclear factor-kB
NLRP3: NOD-, LRR- and pyrin domain-containing protein 3
OB: Obliterative bronchiolitis
PAMPs: Pathogen-associated molecular patterns
PGD: Primary graft dysfunction
PBMC: Peripheral blood mononuclear cell
PRRs: Pattern recognition receptors
qPCR: Quantitative polymerase chain reaction
SGRQ-C: St. George’s Respiratory Questionnaire
TLRs: Toll-like receptors
UFP: Ultrafine particle
UTP: Uridine triphosphate
