# Supplementary material for: Assessment of NLRP3 inflammasome activation in patients with chronic obstructive pulmonary disease before and after lung transplantation
Source: Immunol Res. 2024 May 29;72(5):964–74. doi: 10.1007/s12026-024-09497-2 (PMC11564204; doi:10.1007/s12026-024-09497-2)
Supplement: Supplementary file 1 — Supplementary Material 1 [file 12026_2024_9497_MOESM1_ESM.docx]

**A list of the supplemental materials:**

**Table S1.** Basic characteristics and spirometry parameters of healthy subjects and COPD patients from our larger research.

**Table S2.** Concentrations of eHsp70, eATP and IL-1β in plasma of healthy control group and COPD patients group from our larger research.

**Table S3.** Relative expression of genes encoding eHsp70 receptors and eATP receptors in healthy control group and COPD patients group from our larger research.

**Table S4.** Relative expression of genes encoding IL-1β and NLRP3 inflammasome components in healthy control group and COPD patients group from our larger research.

**Supplementary data**

**Table S1.** Basic characteristics and spirometry parameters of healthy subjects and COPD patients from our larger research.

| **parameter** | **controls**  (n = 95) | **COPD patients**  (n = 109) | **P-value** |
| --- | --- | --- | --- |
| **age** | 64 (46 – 83) | 65  (45 – 87) | 0.069 |
| **sex** male female | 49  46 | 69  40 |  |
|  |  |  | 0.121 |
| **smoking status**  current smokers  former smokers  never smokers | 47  0  48 | 29  75  5 | <0.001 |
| **FEV_1_ (L)** | 2.60 (2.12 – 3.19) | 1.08 (0.69 – 1.60) | <0.001 |
| **FVC (L)** | 3.35 (2.77 – 4.16) | 2.28 (1.74 – 2.77) | <0.001 |
| **FEV_1_ (% pred.)** | 93.3 (86.4 – 104.2) | 40.8 (27.9 – 61.7) | <0.001 |
| **FEV_1_/FVC (%)** | 80.6 (76.8 – 87.6) | 51.3 (40.7 – 58.7) | <0.001 |

FEV_1_ – forced expiratory volume in the first second; FVC – forced vital capacity.

Age is presented as median with minimum and maximum, and sex is presented as an absolute number. Other parameters were shown as median with interquartile range. Data was analysed by Chi-squared or Mann-Whitney test. Results were statistically significant when P < 0.05.

**Table S2**. Concentrations of eHsp70, eATP and IL-1β in plasma of healthy control group and COPD patients group from our larger research.

| parameter | controls  (n = 95) | COPD patients  (n = 109) | P-value |
| --- | --- | --- | --- |
| **eHsp70 (ng/mL)** | 0.98  (0.63 – 1.29) | 0.37  (0.25 – 0.63) | < 0.001 |
| **eATP (µM)** | 0.88  (0.70 – 1.07) | 1.88  (1.26 – 2.89) | < 0.001 |
| **IL-1β (pg/mL)** | 0.10 (0.10 - 0.61) | 6.90 (0.61 – 23.91) | < 0.001 |

eHsp70 – extracellular heat shock protein 70; eATP – extracellular adenosine triphosphate; IL-1β – interleukin 1β.

Data were analysed by Mann-Whitney test and shown as median with interquartile range. Results were statistically significant when P < 0.05.

**Table S3.** Relative expression of genes encoding eHsp70 receptors and eATP receptors in healthy control group and COPD patients group from our larger research.

| parameter | controls  (n = 95) | COPD patients  (n = 109) | P-value |
| --- | --- | --- | --- |
| ***TLR2*** | 1.03  (0.70 – 2.65) | 1.89  (0.87 – 8.41) | 0.010 |
| ***TLR4*** | 0.72  (0.56 – 0.94) | 0.74  (0.56 – 0.98) | 0.780 |
| ***P2X7R*** | 2.08  (1.75 – 2.73) | 2.07  (1.56 – 2.81) | 0.662 |
| ***P2Y2R*** | 4.65  (2.90 – 15.56) | 10.49  (5.26 – 64.23) | < 0.001 |

*TLR2* – Toll-like receptor 2; *TLR4* – Toll-like receptor 4; *P2X7R* – P2X7 purinergic receptor; *P2Y2R* – P2Y2 purinergic receptor.

Data were analysed by Mann-Whitney test and shown as median with interquartile range. Results were statistically significant when P < 0.05.

**Table S4.** Relative expression of genes encoding IL-1β and NLRP3 inflammasome components in healthy control group and COPD patients group from our larger research.

| parameter | controls  (n = 95) | COPD patients  (n = 109) | P-value |
| --- | --- | --- | --- |
| ***IL1B*** | 0.50  (0.34 – 0.78) | 0.66  (0.49 – 1.01) | 0.001 |
| ***NLRP3*** | 1.16  (0.99 – 1.54) | 1.63  (1.31 – 1.92) | < 0.001 |
| ***CASP1*** | 1.27  (1.05 – 1.60) | 1.71  (1.29 – 2.37) | < 0.001 |

*IL1B* – interleukin-1𝛽; *NLRP3* – NOD-, LRR- and pyrin domain-containing protein 3; *CASP1* – caspase-1.

Data were analysed by Mann-Whitney test and shown as median with interquartile range. Results were statistically significant when P < 0.05.
